# Quantifying the Chemical Composition and Real-Time Mass Loading of Nanoplastic Particles in the Atmosphere Using Aerosol Mass Spectrometry

**DOI:** 10.1021/acs.est.3c10286

**Published:** 2024-02-08

**Authors:** Sining Niu, Ruizhe Liu, Qian Zhao, Sahir Gagan, Alana Dodero, Qi Ying, Xingmao Ma, Zezhen Cheng, Swarup China, Manjula Canagaratna, Yue Zhang

**Affiliations:** † Department of Atmospheric Sciences, Texas A&M University, College Station, Texas 77843, United States; ‡ Environmental Molecular Sciences Laboratory, Pacific Northwest National Laboratory, Richland, Washington 99354, United States; § Department of Civil and Environmental Engineering, Texas A&M University, College Station, Texas 77843, United States; ∥ Aerodyne Research Inc., Billerica, Massachusetts 01821, United States

**Keywords:** nanoplastic particles (NPPs), aerosol mass spectrometer (AMS), polystyrene (PS), positive matrix factorization (PMF), multilinear engine (ME-2), real-time measurement

## Abstract

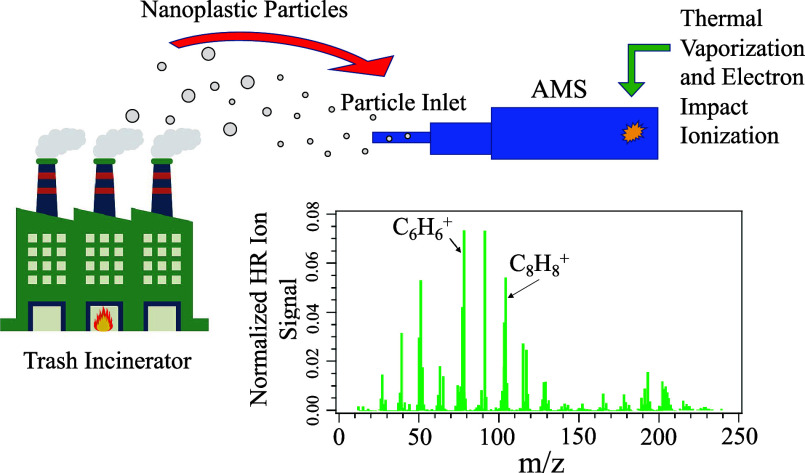

Plastic debris, including nanoplastic particles (NPPs), has emerged as an important global environmental issue due to its detrimental effects on human health, ecosystems, and climate. Atmospheric processes play an important role in the transportation and fate of plastic particles in the environment. In this study, a high-resolution time-of-flight aerosol mass spectrometer (HR-ToF-AMS) was employed to establish the first online approach for identification and quantification of airborne submicrometer polystyrene (PS) NPPs from laboratory-generated and ambient aerosols. The fragmentation ion C_8_H_8_^+^ is identified as the major tracer ion for PS nanoplastic particles, achieving an 1-h detection limit of 4.96 ng/m^3^. Ambient PS NPPs measured at an urban location in Texas are quantified to be 30 ± 20 ng/m^3^ by applying the AMS data with a constrained positive matrix factorization (PMF) method using the multilinear engine (ME-2). Careful analysis of ambient data reveals that atmospheric PS NPPs were enhanced as air mass passed through a waste incinerator plant, suggesting that incineration of waste may serve as a source of ambient NPPs. The online quantification of NPPs achieved through this study can significantly improve our understanding of the source, transport, fate, and climate effects of atmospheric NPPs to mitigate this emerging global environmental issue.

## Abstract 2

Atmospheric plastic particles play important roles regarding the source, transport, and removal of environmental plastic debris. This study establishes a real-time method for quantifying atmospheric nanoplastic particles and demonstrates trash incineration being a potential source.

## 1 Introduction

Micro- and nanoplastics (MNPs), defined as plastic particles with a diameter between 1 μm and 5 mm and below 1 μm, respectively, have been an emerging environmental concern in the global ecosystem.^1−3^ Microplastic particles (MPPs) were first identified as plastic debris in the environment, but subsequent studies showed that nanoplastic particles (NPPs) have distinctive physicochemical behavior and biological interactions due to their smaller sizes. Both MPPs and NPPs (herein referred to as the MNPs) can be classified as either primary or secondary plastic particles based on their sources.^4^ Primary MNPs are plastic products emitted directly into the ambient environment,^5^ and secondary MNPs are the decomposition fragments of larger scale plastic waste.^3^ After being release into the environment, large plastic debris may undergo different transformations related to natural or anthropogenic activities, including aggregation, chemical degradation, and interaction with microorganisms,^6,7^ to form MNPs. It has been shown that MNPs are emitted to the ambient environment during all stages of their lifecycle, from production to usage and waste treatment.^8^ Aside from the adverse impacts of these plastic particles to the ecosystems, previous studies have also demonstrated that airborne plastic particles may also affect climate by enhancing the ice nucleation efficiency and, therefore, cloud formation.^9,10^ In addition, MNPs are shown to negatively impact human health as they can enter the food chain and human body through inhalation^11^ and ingestion.^12^ Compounds made of plastics have already been detected in human placental tissue^13^ and bloodstream^14^ and can cause inflammatory reactions and oxidative stress.^15,16^

Due to the high demand for plastic materials, MNPs have been widely spreaded globally, including but not limited to the marine environment,^17^ freshwater,^18^ land and soil,^19^ and atmosphere.^2^ Having a large volume and being the final reservoir for surface water runoffs, the marine environment has been an important sink for the MNPs.^20,21^ However, studies examining the plastic cycle and plastic transportation among different environmental matrices demonstrate that atmospheric transportation also plays a crucial role in the spreading and fate of MNPs.^8,22^ The atmospheric transportation of plastic materials is relatively rapid compared with other processes,^22^ which facilitate the long-range movement of plastic from their sources to remote areas.^23−27^

To date, various offline chemical analysis techniques have been used to passively or actively sample and analyze atmospheric MNPs.^2^ The collected samples often require additional sample preparation, including but not limited to preconcentration, organic matrix removal, and density separation.^2,28,29^ The prevailing analytical methods include visual identification with microscopy,^18^ thermochemical methods utilizing pyrolysis–gas chromatography–mass spectrometry (pyrolysis-GC/MS),^30^ and vibrational spectroscopy using Fourier-transform infrared (FTIR) spectroscopy^31^ or Raman spectroscopy,^32^ all of which are offline methods that require days to weeks of sampling.^31,33^ It is worth noting that even though most of the thermochemical methods requires pretreatment of the samples, a recent study shows that collected filter samples can be analyzed directly with pyrolysis-GC/MS without pretreatment due to the low matrix natural for atmospheric samples.^34^ Aside from often needing pretreatments, many studies utilizing the methods described above are also constrained by the size of the plastic particles. The size limitations for analyzing plastic fragments are estimated to be 500 μm for visual methods,^35^ 20 μm for FTIR,^31^ and 10 μm for Raman spectroscopy, respectively.^32,36^ For thermochemical methods, the traditional size limit for analyzing plastic particles was suggested to be 100 μm to obtain a clear result.^2^ However, there have been significant improvements with pyrolysis-GC/MS that can identify and quantify lab-generated and ambient plastic particles with much smaller size.^34,37,38^ Considering these size constraints, previous studies have focused more on microplastics with sizes ranging from 5 mm to 1 μm.^2,21^ NPPs have smaller sizes, higher cell affinity, and enhanced surface curvature, making them easier to penetrate into freshwater biological barriers and accumulate in organs than MPPs.^39−42^ Despite their longer atmospheric resident time, enhanced accumulation in the environment, and adverse health effects, nanoplastic particles remain largely uninvestigated due to their small sizes.^2,8,43^ Hence, it is imperative to identify the chemical composition and mass concentration of nanoplastic particles to accurately assess their potential climate effects and public health risks.^44^

Herein, this study demonstrates a real-time online measurement to characterize atmospheric submicrometer polystyrene (PS) NPPs. Polystyrene is top five most abundant plastic compositions produced^2,45^ and identified in the marine system.^46,47^ To the best of our knowledge, this study is the first online method to measure real-time nanoplastics both in the laboratory and in the ambient environment using a high-resolution time-of-flight aerosol mass spectrometer (HR-ToF-AMS). A calibration curve was first established by sampling standard monodispersed PS particles under laboratory conditions. Our results demonstrated that the mass spectra of PS particles can be successfully separated from a complex mixture of inorganic and organic aerosol populations through multivariate factor analysis. A tracer ion of PS NPPs, C_8_H_8_^+^, was then identified by combining laboratory results with ambient measurements. Finally, the mass concentration of atmospheric PS nanoplastic particles was quantified from ambient samplings, with back trajectory analysis suggesting that trash incineration is a potential source. These results are expected to further improve our understanding of the source, evolution, and fate of atmospheric nanoplastic particles.

## 2 Materials and Methods

### 2.1 Working Principle of the Aerosol Mass Spectrometer (AMS)

The high-resolution time-of-flight aerosol mass spectrometer (HR-ToF-AMS) combines a standard vacuum mass spectrometer and aerosol sampling techniques for quantitative measurements of airborne particular matter, with detailed operation principals described in Supporting Information Section S1.^48−50^ Briefly, the airborne particles are focused into a narrow beam through the aerodynamic lens at the inlet and transported into the vacuum, while the gas molecules were deflected. The particles are then directed to a tungsten vaporizer, where the nonrefractory particles are flash-vaporized upon collision. An electron impact (EI) ionizer is employed to ionize the organic molecules into ions, which are then detected by a time-of-flight mass spectrometer.^48−50^ The high-resolution AMS allows direct separation of ions with the same nominal *m*/*z*, and the quantification is possible due to the reproducibility of EI ionization, similar efficiency for all nonrefractory species, and little matrix effect.^51^ The HR-ToF-AMS (herein referred to as AMS) has been widely used in the atmospheric aerosol sciences,^48−51^ and it was employed in all the laboratory experiments and ambient sampling of this study.

### 2.2 Laboratory-Generated PS Particles and Organic/Inorganic Aerosol Mixtures

To obtain the mass spectra of the pure PS NPP standards, an aqueous solution containing 500 nm monodispersed PS particles (Millipore Sigma, Part Number MFCD00243243) was aerosolized with a constant output atomizer (TSI, Model 3076). The size of 500 nm was carefully selected as this size falls within the accumulation mode, where particles typically have longer atmospheric lifetime compared with those in Aitken mode or coarse mode.^52^ Given that AMS evaporates all nonrefractory submicrometer particles efficiently, 500 nm PS particle standards can represent typical NPPs and thus were used for all the lab experiments in this study. A customized silica gel diffusion dryer was used to remove the excess water prior to directing the PS particles to the AMS. The particle mass spectra were collected as a function of time.

To further characterize PS NPPs by mimicking the externally mixed particles in the ambient environment, both inorganic and secondary organic aerosols (SOAs) were generated and externally mixed with PS NPPs of selected ratios. The inorganic aerosols, including ammonium nitrate (Sigma-Aldrich, ≥98% purity) and ammonium sulfate (Sigma-Aldrich, ≥99% purity), were dissolved in Milli-Q water (mass fraction of 0.1%) and then atomized with a collision nebulizer (CH Technologies, USA). The SOAs were generated from the oxidation of selected volatile organic compound (VOC) precursors with ozone or OH radical in a potential aerosol mass (PAM) oxidation flow reactor (Aerodyne Research, Inc.).^53,54^ Both biogenic (α-pinene, Sigma-Aldrich, 98% purity) and anthropogenic (toluene, Sigma-Aldrich, ≥99.5% purity) VOCs were used to generate SOA so as to obtain a more realistic scenario of nanoplastic particles mixed with other types of aerosols in the ambient environment. The normalized number and mass distributions of the SOA measured by the scanning electrical mobility spectrometer (SEMS, Brechtel, model 2100) are shown in Figure S1. The details of PAM operations are listed in Supporting Information Section S1. Briefly, the VOC precursor was injected into a three-necked manifold at constant rate by a syringe pump (Chemyx, Model Fusion 400) and carried out by 1 L per minute (LPM) flow of zero air. The ozone was generated through an in-house customized ozone generator at a flow rate of 1.5 LPM. The reaction of the VOC with ozone in the PAM produced homogeneously nucleated SOA particles, which were then mixed with PS NPPs downstream before being analyzed by the AMS. The detailed flowchart and schematic diagram of the experimental setup are shown in Figure S2. Each reaction condition was repeated with five different mixing ratios of PS NPPs and laboratory-generated aerosols by adjusting the flow rates of two respective lines. The atomizer, sampling line, and PAM were flushed with deionized water, dry air, and ozone between experiments to prevent potential contaminations after each set of experiments. The mass spectra were collected with the AMS in *V* mode, and the data were analyzed with the Squirrel (version 1.65) and Pika (version 1.25) packages in Igor Pro (WaveMetrics Inc., version 8.04).

### 2.3 Ambient Sampling

To quantify the PS NPPs and their potential sources in the atmosphere, ambient particle sampling was conducted twice at the Texas A&M University, College Station, TX, which are referred to as ambient1 and ambient2 hereafter. The sampling inlet was located at the Eller Oceanography & Meteorology Building and 40 m above ground level to minimize local and surface influence. To further validate the identification and quantification of nanoplastic particles, atomized PS particles were injected intentionally into the ambient environment at a distance of 10 cm from the AMS sampling inlet toward the end of the sampling period during ambient1 and at selected times during ambient2 samplings, with detailed schematic of the setup shown in Figure S3.

### 2.4 Deriving Nanoplastic Mass Concentrations Using Positive Matrix Factorization (PMF) and the Multilinear Engine (ME-2)

The positive matrix factorization (PMF) method was applied to the time-series mass spectral data to separate the PS particles from inorganic aerosols and SOAs. PMF is a multivariate factor analysis model that has been widely used in source apportionment of atmospheric components.^55^ Specifically, PMF has been applied to aerosol mass spectrometry for factor separation by decomposing the mass spectra and signal of the measured aerosol population with a linear combination of various factors, with detailed working principles of PMF described in Supporting Information Section S2 and brief operational procedures described below.^56−58^ The isotope-excluded data and error matrices for PMF were first generated in the analysis software Pika. With a selected solution, the minimum summation of the weighted square residuals, *Q*, was calculated. Normalizing the *Q* value with the expected *Q* value (*Q*_exp_), defined as a function of the size of the data matrix and the number of the factors, was used as a key parameter to evaluate the results.^57^ The PMF analysis was performed with Igor PMF version 3.07.

To quantify the nanoplastic particles with low mass concentration from ambient atmospheric environments, the bilinear model of the multilinear engine (ME-2),^59^ which allows the use of constrained factor profiles, was applied to the collected mass spectra. Compared with unconstrained PMF, ME-2 with a pre-existing input mass spectral profile will direct the bilinear model toward an optimal solution under certain situations where PMF underperforms. Generally, ME-2 is particularly suited for scenarios with factors that have similar temporal variation, relatively low concentration, and high rotational ambiguity.^60^ Ambient airborne NPPs are suitable for ME-2 analysis due to their low concentration in the environment. In the ME-2 analysis, the constraint factor, namely, the *a* value, has been utilized in previous studies to define the AMS mass spectra for a specific factor.^60−62^ The *a* value determines the extent to which the derived factor profile could vary from the input spectrum profile. The software package for ME-2, SoFi (Source Finder),^60^ was used to identify ambient PS NPPs, and the detailed description of ME-2 is shown in Supporting Information Section S3.

## 3 Results and Discussion

### 3.1 Chemical Characterization of Pure PS Nanoplastic Particles

**Figure 1:**
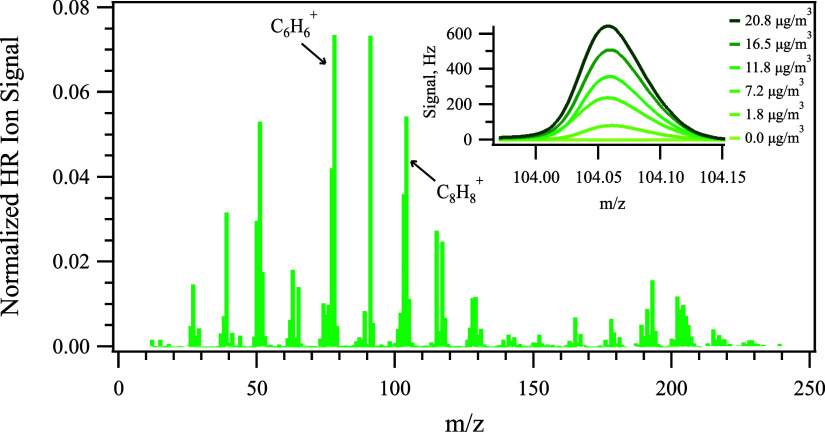
HR-ToF-AMS organic mass spectra for pure PS particles. Inset: the gaussian fit for tracer ion C_8_H_8_^+^ under different mass concentrations of PS NPPs, which were indicated by the legend, during the calibration.

The high-resolution mass spectrum of PS NPP was collected by the AMS, as shown in Figure 1. The mass spectrum was dominated by C_*x*_H_*y*_, with a ratio of 93 ± 3% by mass. As PS is made of long chains of ethenylbenzene monomers (C_8_H_8_), electron ionization fragments the long chains and generates C_*x*_H_*y*_. There were also minor fragmentation ions in the AMS mass spectra containing oxygen and nitrate (<2%), which could come from either the oxidation of PS particles or the heated vaporizer region of the AMS. Among the C_*x*_H_*y*_ family, C_6_H_6_^+^, C_7_H_7_^+^, and C_8_H_8_^+^, corresponding to mass-to-charge ratios (*m*/*z*) of 78, 91, and 104 in unit mass resolution (UMR), were the three major ions in the PS mass spectrum. Given that organic species in atmospheric aerosols often fragment in the AMS to form ions with odd mass-to-charge ratios (except for nitrogen-containing compounds),^63^ ions with even mass-to-charge ratios from the PS mass spectrum, such as *m*/*z* 78 and 104, are potentially unique to PS-containing particles and are further discussed in Section 3.4.

**Figure 2:**
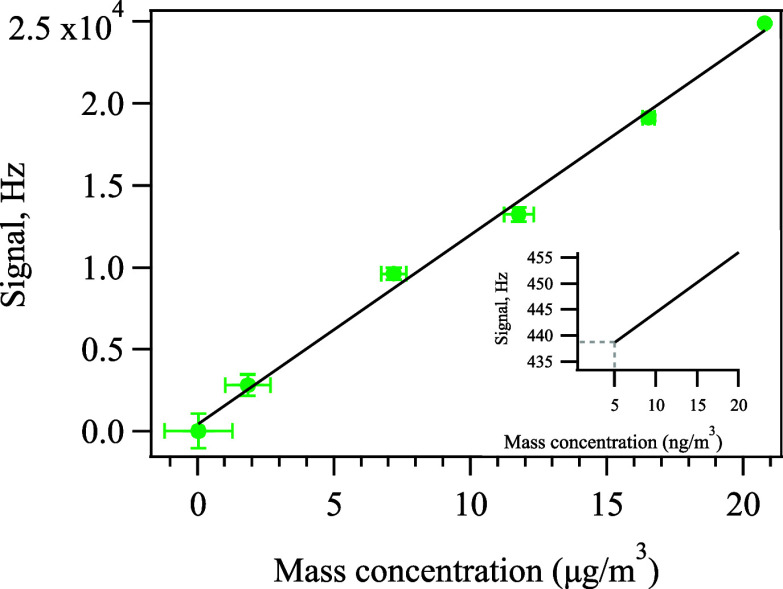
Calibration curve of PS particles with a slope value of 1.155 × 10^3^ Hz/(μg/m^3^) and *R*^2^ = 0.995. The *x*-axis is the mass concentration of PS particles calculated based on the CPC measurements, and the *y*-axis shows the organics signal measured by the AMS. Inset: the vertical dashed line represents the detection limit calculated based on the calibration curve, while the horizonal dashed line represents the 3σ of the noise averaged over 1-h period.

In aerosol mass spectrometry, ionization efficiency (IE), which reflects the number of ions detected per molecule sampled, is a key term in quantifying the absolute mass of the compound.^50^ In addition to an IE calibration with nitrate,^49,50^ an analogous calibration for PS particles was carried out with an AMS and a mixing condensation particle counter (CPC, model 1720, Brechtel). The signal of total organic concentration obtained by the AMS versus the mass concentration of PS particles derived from the CPC is shown in Figure 2, demonstrating the accurate calibration of PS particles. By assuming a collection efficiency (CE) of 1, which is adopted from ammonium nitrate particles between 100 and 1000 nm,^48,64^ the IE of the PS is calculated to be 1.155 × 10^3^ Hz/(μg/m^3^), corresponding to the slope shown in Figure 2. Based on the calibration data, the detection limits for PS particles with AMS are calculated to be 12 and 5 ng/m^3^ for 10 min and 1 h of sampling, respectively, derived from three times the standard deviation of blank signals when a filter was installed at the AMS inlet.^65^ Such low detection limits enable real-time quantification of ambient nanoplastic particles using the AMS. The RIE of PS particles is 1.88 with respect to nitrate assuming a unity CE, with detailed calculations and mass spectra shown in Supporting Information Section S4 and Figures S4–S7.

To further validate the quantification of PS NPPs with the AMS, a pyrolysis-GC/MS analysis was also conducted on the same samples analyzed by the AMS, with details discussed in Supporting Information Section S5 and Figure S8. Briefly, the PS NPP standards were collected by both the AMS and glass fiber filters (Cytiva, 0.7 μm particle retention) simultaneously. The mass loadings quantified by pyrolysis-GC/MS and AMS agree reasonably well within 2% of uncertainty. The above validation further confirms accurate quantifications of PS NPPs by the AMS.

### 3.2 PS Factor Extracted from the Mixtures of Laboratory-Generated Aerosols

To mimic complex ambient aerosols mixed with PS nanoplastic particles, binary systems consisting of selected types of inorganic aerosols and SOAs externally mixed with PS particles in a mixing tube are analyzed by the AMS. The PS NPPs are identified and quantified using the PMF as described in Section 2. As indicated by the PMF results, more than 99% of the total mass could be well explained by the two aerosol sources (i.e., two factors) with the scaled residual (*Q*/*Q*_exp_) dropping significantly with two factors. The diagnostic plots, including *Q*/*Q*_exp_ and scaled residual for the ammonium nitrate mixture, are shown in Figure S9 as an example. These two-source results from the PMF analysis on the mixture mass spectra are consistent with the experimental setup, where two lines of aerosols were mixed. In addition, as the ratio of the flow rates from the two aerosol sources was changed, PMF results also successfully captured the variations of the concentration of different factors, as indicated by the time series of the two factors in Figure S10.

**Figure 3:**
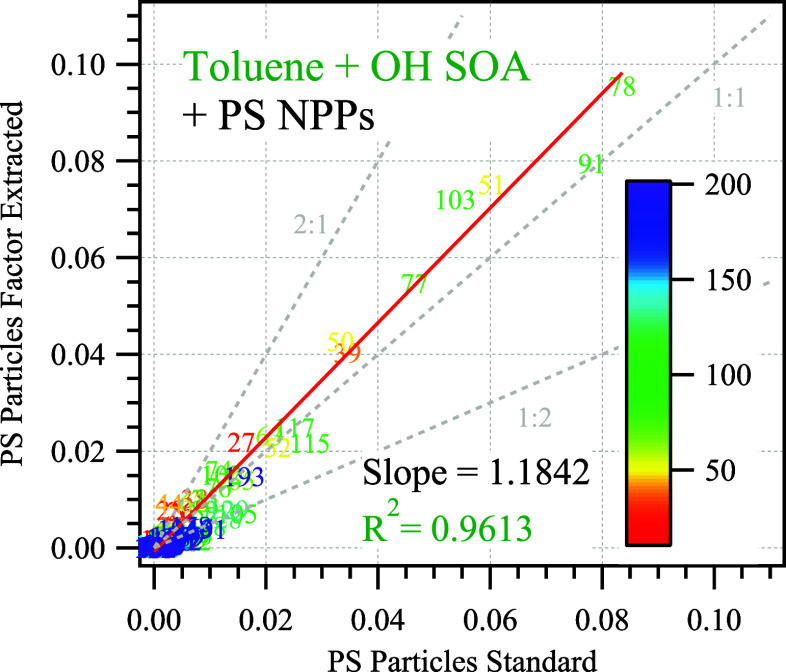
Scatter plot of normalized HR organic ion signal of PS particles from the factor extracted from PMF (*y* axis) versus the pure standard (*x* axis) for the binary mixture system containing toluene SOA and PS particles. The numbers are the *m*/*z* from the mass spectra. The color bar represents the values of the *m*/*z* from the mass spectrum in ascending order.

The normalized spectra for the PS NPP factor derived from the mixture using the PMF method can reproduce the spectrum for pure PS particles with an averaged coefficient of determination *R*^2^ = 0.95, as shown in Figure 3 and Figure S11. The correlation slopes between the mass spectrum derived from the PMF and the mass spectrum of the PS particle standards vary between 1.06 and 1.20 depending on the composition of the mixture. Careful examination of the correlation data suggests that the mass spectra of the PS NPP factor from the organic mixtures are less dominated by *m*/*z* 104 compared with the standards. The difference of *m*/*z* 104 suggests that a small fraction of other ions from the SOAs might be partially assigned to the PS NPP factor, causing the ratio of *m*/*z* 104 to the whole mass spectra to be lower and the slopes of the correlation plots in Figure 3 and Figure S11 to be slightly higher than unity. The uncertainty for characterizing PS from a mixture of lab-generated aerosol particles with PMF is estimated to be around 20%, calculated from the deviation of the correlation slopes from unity as shown in Figure 3 and Figure S11. The above results successfully demonstrate that PS nanoplastic particles could be separated from common atmospheric aerosols by using the PMF factor extraction method with an uncertainty range of 20% .

### 3.3 Concentration of Ambient Airborne PS Nanoplastic Particles

In this study, ambient air was also sampled by an AMS to quantify the atmospheric PS NPPs. Due to the much lower concentration of PS nanoplastic particles compared with the laboratory experiments, the ME-2 method was applied to the dataset to improve the factor extraction and subsequent quantification. The ME-2 algorithm only constrains the mass spectra of PS nanoplastic particles while allowing self-identification of other factors. The final solution was carefully chosen based on the correlation of the profiles as well as time series information on the retrieved factors, the relative residual, and the tracer ions.^60,62^

**Figure 4:**
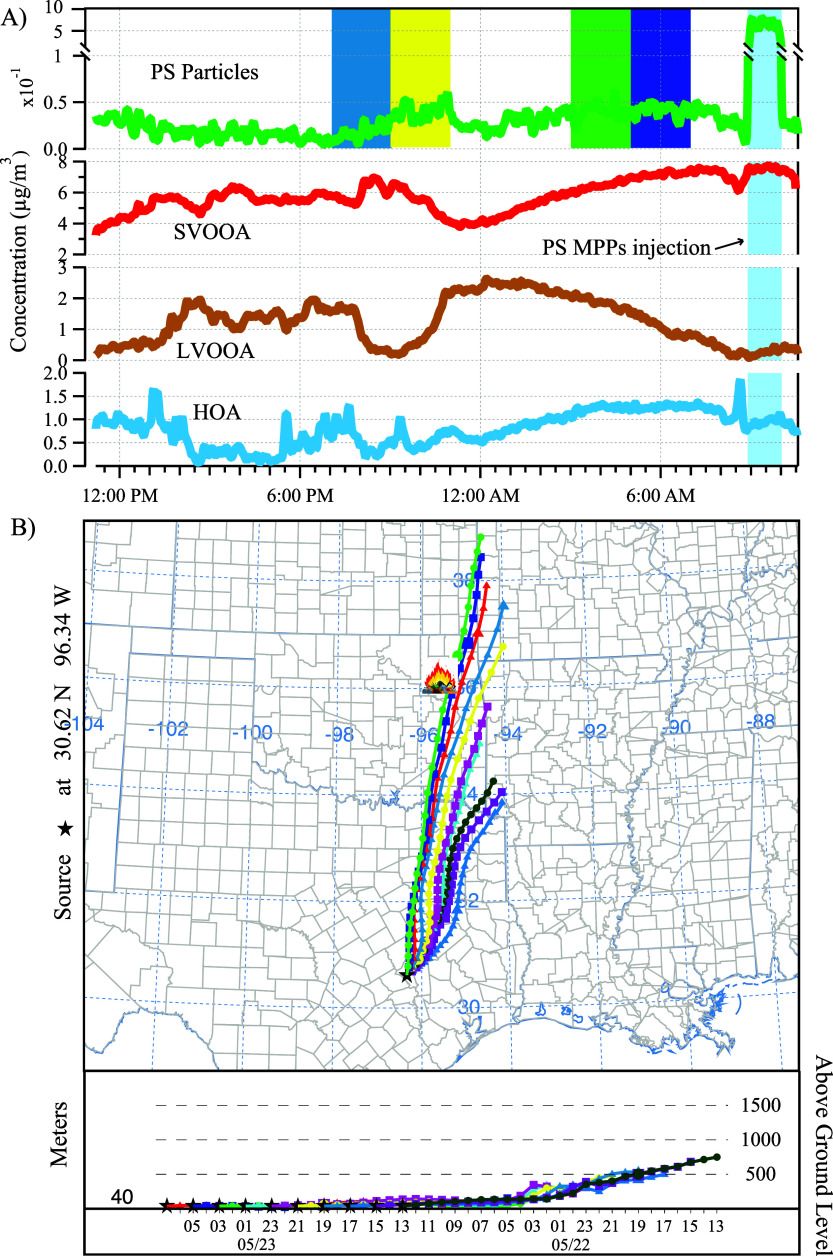
The time series of each factor identified during ambient1 from the ME-2 analysis (A), and the 24 h backward trajectories of air masses arriving at the sampling inlet (B) at 40 m above ground level during ambient1. The trajectories were retrieved every 2 h to elaborate the trend of derived PS factor. In Panel A, the four intervals during which the backward trajectories passed in close proximity to the waste incinerator were highlighted with the same color as their corresponding trajectories in Panel B. The legend and air mass height at the bottom of Panel B show the arrival time of the air mass at the sampling site every 2 h. These four intervals showed elevated nanoplastic concentrations.

After performing ME-2 analysis, the best ME-2 solution for the ambient1 sampling period had 4 factors, with each of the time series and mass spectra shown in Figure 4A and Figure S12, respectively. The first factor is constrained by the pure PS mass spectrum, corresponding to the PS NPPs. Two types of OOA, i.e., more-oxidized OOA (MO-OOA) and less-oxidized OOA (LO-OOA), are identified in ambient1 sampling. The factor corresponding to MO-OOA showed a higher O:C ratio and *f*_44_ compared with LO-OOA, agreeing with previous literature results.^66,67^ Moreover, the time series of MO-OOA and LO-OOA are uncorrelated, suggesting that they may be from different air mass with different sources.^66,67^ The fourth factor represents the hydrocarbon-like organic aerosol (HOA) with the highest H:C ratio and lowest O:C ratio of all nonplastic factors. The HOA factor here also matches with those reported from previous literature with relatively higher signal intensities at *m*/*z* 55 (C_4_H_7_^+^) and 57 (C_4_H_9_^+^), which have been identified as markers of fresh fossil fuel combustion.^68−70^ The correct assignment of plastic and other non-plastic factors further validate the ME-2 analysis results and demonstrate that ME-2 can be used for source apportionment of aerosol composition including nanoplastic particles. The diagnostic plots in Figure S13 show the scaled residual as a function of time and *m*/*z*. The 10 to 90 percentiles of the scaled residual lie between ±3, further validate support the assignment of factors from the ME-2 results.^71^ The results of ambient2 are similar to those of ambient 1, with the details described in Supporting Information Section S6 and Figure S14.

As discussed in Section 2.3, PS NPPs were injected near the sampling inlet at selected periods of sampling to validate the quantification of the AMS. During the period when PS NPPs were released into the air near the sampling inlet (blue shaded area in Figure 4A), factor 1, representing the PS NPPs, was the only factor that showed a significant enhancement of its measured concentration, while the total organic concentration (Figure S14) and all other factors remained stable. The stability of other factors further validates that only factor 1 represents the concentration of ambient PS NPPs. To verify the reliability and reproducibility of the ME-2 results, two sensitivity tests were performed to determine the level of constraint to the reference profile of PS particles. For the first sensitivity test, we construct the *a* value of the PS NPP factor from 0.2 to 0.7, resulting in averaged concentrations of PS particles fluctuated by 16%, suggesting that the factors derived for PS nanoplastic particles are generally stable and robust despite changing *a* values.^60,62,72,73^ For the second sensitivity test, a series of synthetic organic aerosol data containing known concentrations of PS NPPs are analyzed with ME-2. The time series of PS NPPs ranging from 16.2 to 333 ng/m^3^ were synthetically added to the time series of organic aerosol mass spectra collected from ambient measurement with PS NPP profile removed. The concentrations of the PS NPP derived from this synthetic data set using the ME-2 analysis agree with the prior-known concentrations of the PS NPPs added to the spectra, as shown in Table S1. Furthermore, ME-2 analysis could successfully and accurately extract hidden PS NPP concentrations as low as 16.2 ng/m^3^, a value even below the ambient concentration of PS NPPs measured from this study. Such results further validate accurate source apportionment of airborne polystyrene nanoplastic particles via online mass spectrometry coupled with the ME-2 analysis down to tens of nanograms. The detailed sensitivity analysis procedures are shown in Supporting Information Section S7.

The average concentration of PS NPPs in the ambient environment was estimated to be 30 ± 20 ng/m^3^ based on the time series data of the ME-2 results and excluding the time periods when PS NPPs were artificially introduced into the air. To date, most studies that quantify atmospheric microplastics have relied on passive collection, followed by microscopic analysis. The concentration of different types of total MNPs quantified in previous studies can be estimated to ranges from 2 to 8 × 10^9^ ng/m^3^,^25,74−77^ spanning 9 orders of magnitude regarding the mass of deposited or suspended airborne microplastics, with detailed procedures for such estimation shown in Supporting Information Section S8.^2^ In general, the more polluted the environments are, the more airborne microplastics that were identified. However, variations of concentrations among different sites occur and can be attributed to either different background environments or the size ranges targeted by each study. The wide range and large fluctuations observed from past measurements underscore the importance of an online quantitative analytical method for atmospheric nanoplastic particles. The derived concentration of PS nanoplastic particles in our sampling site is on the lower end of the estimated concentration range listed above, which is reasonable considering that our site is in a relatively clean rural area, as indicated by the low total organic aerosol concentration. Long-term monitoring of atmospheric NPPs using the AMS for a wider range of locations is desired to examine their spatial and temporal trends and to understand their impacts on the environment.

The use of an online real-time mass spectrometer can also provide accurate concentrations of atmospheric NPPs for examining the human exposure and other health effects of plastic materials. MNPs have been shown to cause neurotoxicity,^78^ inflammation,^15,16^ DNA damage,^78,79^ alternation of gene expression,^80^ etc.^81^ Based on the averaged concentration of ambient airborne NPPs derived in this study and an inhalation rate of 11 m^3^/day,^82^ the exposure of an adult to PS NPPs in College Station derived from this measurement is estimated to be at the order of 100 mg/year. This extrapolated annual concentration of PS NPPs assumes that the conditions are the same for indoor and outdoor environments regardless of seasonal change and thus may provide a rough estimation of potential atmospheric nanoplastic exposure that may be useful in its magnitude. In addition, the exposure calculated may skew toward the lower end due to the finding that higher concentrations of airborne microplastics were found to be present in the indoor environment than the outdoor environment.^83,84^ The *in vivo* inhalation toxicity of PS NPPs was evaluated using a whole-body inhalation system with rats as the experimental animal.^85^ Under different PS NPP exposures ranging from 22 to 100 μg/m^3^ for 14 days, serum biochemistry, pulmonary function, bronchoalveolar lavage, lung tissue, and Western blot analyses of the rats were performed. The results suggested that PS NPP exposure has a distinct effect at the molecular level by increasing the inflammatory protein expression, and there exists the potential health risk at a higher level (e.g., organismal level) if the exposure is sustained.^85^ Future studies are needed to examine the long-term exposure of atmospheric NPPs at ambient levels. The toxicity and concentration of PS NPPs above highlight the critical need for further research aimed at quantifying the toxicity of atmospheric nanoplastic particles with regard to their potential impacts on human health.

Other than the source apportionment bilinear model ME-2, the Hybrid Single Particle Lagrangian Integrated Trajectory model (HYSPLIT) was also applied to the stationary ambient sampling to assess the atmospheric trajectory and potential source of the PS NPPs.^86^ The 24 h back trajectories of the air mass at the sampling inlet were evaluated with a 2 h time interval, and they are shown in Figure 4B. In general, during the 20 h sampling period, the measured air mass was consistently from the north of the College Station, with the altitude gradually decreasing from 800 m above ground level to 40 m at the sampling site. As indicated by the illustration in Figure 4B, a trash incineration site is located near the back trajectory pathways, and it is the only municipal waste combustor in the surrounding area with 420 miles radius from all of the back trajectories. A careful analysis of the back trajectories of the air mass shows that when the wind direction is the closest to the trash incineration site, i.e., May 22, 8 to 11 pm, and May 23, 3 to 8 am, the ambient PS NPP concentration was elevated as indicated by the color-coded time period in Figure 4A. A *t* test was performed for the PS NPP data set, and the concentration during the color-coded time period in Figure 4A shows a statistically significant increase in comparison with that from 4 to 6 pm (*p* < 0.01), with detailed results shown in Table S2. Micro- and nanoplastic particles have been reported to generate from incineration with the Raman spectroscopy method,^87−89^ agreeing with our ambient observation. Previous studies also have shown that nanoplastic particles comprised of both PS and polyethylene terephthalate (PET) can be generated at temperatures as low as 200 °C through homogeneous nucleation when the plastic is melted and cooled.^90,91^ A waste incinerator can often be heated to 650 to 1100 °C, higher than the melting and vapor recondensation onset temperature of 200 °C from which nanoplastics can be generated. In addition, potential incomplete combustion in the incineration of waste further facilitates the generation of byproducts including nanoplastic particles.^92−95^ It is worth noting that the PS NPP concentration decreased significantly between 10 pm to 2 am from March 22–23, suggesting that the PS NPP enhancement intervals before and after this period were not due to the change of the boundary layer height but likely due to external sources of PS NPPs. Other than polystyrene, as many as nine types of airborne NPPs have been reported to be generated from the flying ashes of the trash incinerators.^94,95^ The above correlation between the PS NPP concentration and the passing of air mass through the trash incineration site indicates that the city’s solid waste treatment could be a potential source of atmospheric NPPs.

### 3.4 Tracer Ion of PS Nanoplastic Particles

**Figure 5:**
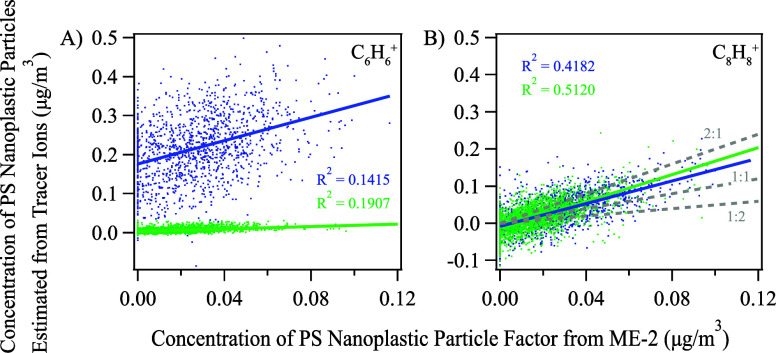
The correlation between the concentration of PS nanoplastic particle derived from tracer ions A) C_6_H_6_^+^, B) C_8_H_8_^+^ (*y* axis) and ME-2 (*x* axis) for ambient sampling (blue: *ambient1*, green: *ambient2*).

In Section 3.1, C_6_H_6_^+^ and C_8_H_8_^+^ were identified as potential tracer ions for PS NPPs from pure nanoplastic spectra and laboratory experiments with externally mixed aerosol particles. With ambient data, we further examined the robustness of these two ions serving as tracers for the PS nanoplastic particles. Figure 5 shows the correlations between the concentrations of C_6_H_6_^+^ and C_8_H_8_^+^ and the PS NPP factor during both ambient sampling periods. The concentrations of PS nanoplastic particles were not correlated to the signals of C_6_H_6_^+^ (*R*^2^ = 0.14 and 0.19 for two sampling periods) but correlated relatively well with the signals of C_8_H_8_^+^ (*R*^2^ = 0.42 and 0.51 for two sampling periods). In addition, the slopes of the C_8_H_8_^+^ signals from two independent field data sets are similar despite two different air mass dominating during these two sampling periods, suggesting that C_8_H_8_^+^ could be a robust tracer ion. In addition, the C_6_H_6_^+^ ion can also come from secondary organic aerosols, such as decomposing aromatics,^96−98^ further suggesting that C_8_H_8_^+^ is a better tracer for identifying PS nanoplastic compounds. It has been reported in previous studies that chitin, a natural biopolymer in water and soil that is likely from crustaceans, insects, and invertebrate animals, can release C_8_H_8_^+^ during pyrolysis.^99−101^ Hence, water and soil samples with complicated environmental matrices encompassing components from the biosphere may cause chitin to contribute to the C_8_H_8_^+^ signal, leading to inaccurate estimation of PS NPPs with C_8_H_8_^+^. However, atmospheric nanoplastic samples are generally low matrices and have less interference from the biosphere, leading to the potentially minimal influences of chitin on the quantification of NPPs.

To examine the feasibility of using tracer ion C_8_H_8_^+^ to identify PS NPPs, the concentrations of PS particles are calculated from both the ME-2 analysis and tracer ion C_8_H_8_^+^ with eq S4. The fraction of C_8_H_8_^+^ in the mass spectrum of pure PS standards was combined with the actual signal of C_8_H_8_^+^ from ambient aerosols to calculate the concentration of atmospheric PS NPPs, with details illustrated in Section S9. The concentration derived from the C_8_H_8_^+^ tracer ion is 25% higher on average than the value derived from the ME-2 method. The results suggest that C_8_H_8_^+^ might overestimate the ambient PS nanoplastic concentration due to other sources of C_8_H_8_^+^ from the aerosols, as stated above. Given that C_8_H_8_^+^ is a reduced alkane fragment, it has not been recognized as a tracer ion for the most common factors extracted by the PMF for ambient aerosols in previous studies^69,70^ and has only been identified in two studies where it was attributed to monoterpene emission^102^ and aromatics.^103^ Other than polystyrene, there exist other types of plastic polymers in ambient NPPs, for instance, polypropylene, polyethylene, and polyvinyl chloride. Due to different chemical structures, their mass spectra from pyrolysis-GC/MS are different.^104^ Hence, it is unlikely for other types of NPPs to cause large uncertainties in quantification of PS NPPs as they will likely not generate the same tracer ions of C_8_H_8_^+^ as PS. Overall, given a lack of techniques in quantifying atmospheric NPPs, tracer ion C_8_H_8_^+^ is still useful in identifying PS NPPs especially when statistic tools such as ME-2 are not available.

### 3.5 Environmental Implications and Applications

This study establishes a real-time online method to quantify PS NPPs for the first time using an aerosol mass spectrometer and identifies the fragmentation ion C_8_H_8_^+^ (*m*/*z* 104) as a potentially tracer ion for identifying and quantifying PS NPPs. The calibration curve of PS NPPs using AMS shows that this online method can achieve low detection limits of 12 and 5 ng/m^3^ over the course of 10 min and 1 h, respectively. Laboratory-generated binary mixtures of inorganic and organic aerosols were successfully separated from the PS NPPs using the PMF technique, with the PMF factor of PS particle successfully identified from the externally mixed aerosol population. The spectra of the PS nanoplastic factor are highly similar to those of the authentic PS standards, with *R*^2^ > 0.95, suggesting that the nanoplastic mass spectra are different from those of SOA particles. Subsequent ambient measurements also derived a PS NPP factor using ME-2 analysis, with the ambient concentration of PS NPP estimated to be 30 ± 20 ng/m^3^. The annual exposure of ambient PS NPP may cause non-negligible and potentially adverse effects on human health. Back trajectory analysis demonstrates that trash incineration may also be a potentially important source of atmospheric NPPs.

For ambient data analysis, ME-2 constrains the whole mass spectrum and thus can successfully identify PS NPPs from a complex atmospheric particle population. The combination of AMS with ME-2 also has the potential to identify and quantify other types of airborne NPPs, as various types of plastics may fragment differently due to their unique molecular composition and structures.^104^ Further studies are needed to verify the feasibility of identifying other types of NPPs using the AMS and to examine the health effects when exposed to ambient levels of nanoplastic particles. In summary, this real-time quantification of ambient nanoplastic particles provides a unique tool to examine the abundance, distribution, life cycle, and health and climate impacts of atmospheric nanoplastic particles, bridging the gap in understanding the role of atmosphere processes in the environmental cycle of micro- and nanoplastic particles.

## Supporting Information Available

Section S1: aerosol mass spectrometry and potential aerosol mass reactor operation conditions; Section S2: positive matrix factorization (PMF) working principle; Section S3: detailed analysis procedures of ME-2; Section S4: relative ionization efficiency (RIE) of polystyrene nanoplastic particles; Section S5: comparison of PS NPP quantification with the AMS and pyrolysis-GC/MS; Section S6: second ambient sampling; Section S7: ME-2 analysis of the synthetic data matrix; Section S8: concentration of MNPs from the literature; Section S9: concentration of PS NPPs derived from tracer ions; Figure S1: number and mass distribution of self-nucleated SOAs generated though PAM; Figure S2: schematic of the experimental setup for the mixing of PS nanoplastic particles and inorganic/organic aerosols; Figure S3: schematic of the ambient sampling setup; Figure S4: correlation heat map of the mass spectra of PS NPPs under different concentrations; Figure S5: mass spectrum of ammonium nitrate collected by the AMS during calibration; Figure S6: mass spectrum collected by the AMS with a filter at the inlet; Figure S7: derived concentration of the tracer ions of PS NPPs with a filter at the inlet of the AMS; Figure S8: mass spectrum of PS NPPs from pyrolysis-GC/MS; Figure S9: scaled residual and *Q*/*Q*_exp_ from PMF for lab experiment; Figure S10: time series of the factors extracted from the binary mixture system; Figure S11: correlation between the mass spectrum for the authentic PS particle standard and the factors extracted by the PMF; Figure S12: mass spectrum of each factor from ME-2 during ambient sampling; Figure S13: scaled residual for ambient sampling; Figure S14: time series of total organics during ambient sampling; Table S1: sensitivity analysis of ME-2 applied on ambient-level concentrations of PS NPPs; Table S2: averaged mass concentration of PS NPPs from ambient measurements and the *p*-value for *t* test (PDF)
