# Supplementary material for: Quantifying the Chemical Composition and Real-Time Mass Loading of Nanoplastic Particles in the Atmosphere Using Aerosol Mass Spectrometry
Source: Environ Sci Technol. 2024 Feb 8;58(7):3363–74. doi: 10.1021/acs.est.3c10286 (PMC10882961; doi:10.1021/acs.est.3c10286)
Supplement: Supplementary file 1 — es3c10286_si_001.pdf [file es3c10286_si_001.pdf]

SUPPORTING INFORMATION

Quantifying the Chemical Composition and Real-time Mass Loading of Nanoplastic Particles (NPPs) in the Atmosphere using Aerosol Mass Spectrometry

Sining Niu[1], Ruizhe Liu[1,†], Qian Zhao[2], Sahir Gagan[1], Alana Dodero[1], Qi Ying[3], Xingmao Ma[3], Zezhen Cheng[2], Swarup China[2], Manjula Canagaratna[4], Yue Zhang[1*]

[1]Department of Atmospheric Sciences, Texas A&M University, College Station, Texas, 77843, USA

[2]Environmental Molecular Sciences Laboratory, Pacific Northwest National Laboratory, Richland, Washington, 99354, USA

[3]Department of Civil and Environmental Engineering, Texas A&M University, College Station, Texas, 77843, USA

[4]Aerodyne Research Inc., Billerica, Massachusetts, 01821, USA

[†]Now at the School of Earth and Atmospheric Sciences, Georgia Institute of Technology, Atlanta, Georgia, 30332, USA

January 2024

Environmental Science and Technology

[*]*Corresponding author:* Yue Zhang, yuezhang@tamu.edu

No. of pages: 28

No. of figures: 14

No. of tables: 2

1. Aerosol Mass Spectrometry and Potential Aerosol Mass reactor operation conditions

The Aerosol Mass Spectrometry (AMS) is the main instrument for collecting the mass spectra of the nanoplastic particles in this study. Due to the aerodynamic lens and the chopper, the AMS samples particles 10 million times more efficient than sampling gases.[1] Other statistical tools, including the PMF, are used to further aid the data analysis of complicated particle mass spectra collected from the AMS and described in Section S2 below.

The Potential Aerosol Mass (PAM) reactor is a horizontal 13 L copper cylindrical chamber (46 cm long × 22 cm inner diameter) operated in continuous flow mode, and could be used to generate Secondary Organic Aerosols (SOA) from Volatile Organic Compounds (VOCs) with either the ozonolysis or photooxidation pathways.[2,3] In this study, the SOA particles are formed in the absence of any seed particles through OH radical or ozone oxidation. Regarding OH radical oxidation, a 1 liter-per-minute (LPM) purified compressed air passing through the deionized water was introduced into the PAM for the humidification. Another 1.5 LPM air flow was introduced into the homebuilt ozone generator and then entered the PAM. The VOC precursors were introduced into the PAM via the syringe pump injection into a three-neck round-bottom flask, which was carried out by a 1 LPM compressed air flow to the PAM. The OH radicals were generated via the photolysis of ozone with UV lamps in the PAM. In addition, 1 LPM flow of ozone was introduced into the PAM and the ozonolysis was conducted under the dry condition. The UV lights in the PAM were turned off and the ozonolysis was the predominated reaction under dark condition to form low volatile oxidation products, which then undergo self-nucleation to form SOA particles.

2. Positive matrix factorization (PMF) working principle

PMF was first introduced in 1994 by Paatero and Tapper,[4] and has been a useful tool for resolving the time series data for mass spectrometer. The fundamental principle in PMF

analysis is that the measured data matrix of mass spectrum could be expressed by the combination of selected number of factors based on mass conservation.[5] The organic data matrix ***org*** with the dimensions $t \times m$ is modelled according to Eq. S1:

$$org_{i,j} = \sum_{p=1}^{P} ts_{ip} ms_{pj} + e_{ij} \qquad \text{(S1)}$$

where P is the number of factors in the solution, $org_{i,j}$ is the signal of ion fragment j at time step i in the organic data matrix, ts_{ip} is the concentration of factor p at the given time step i, ms_{pj} is the fraction of ion fragment j in the mass spectrum for the particular factor p, and e_{ij} is the residual not included by the solution for ion fragment j at time step i. With a given factor number, the PMF solution provides a minimum summation of the weighted squared residuals Q as indicated by Eq. S2 below:

$$Q = \sum_{i=1}^{t} \sum_{j=1}^{m} (e_{ij}/\sigma_{ij})^2 \qquad \text{(S2)}$$

where σ_{ij} is the estimated errors corresponding to the $org_{i,j}$. As the Q value also depends on the dimensions of the data matrix and the number of factors chosen, normalizing the Q value with the expected Q value (Q_{exp}), which is the degree of freedom of the solution, leads to a useful diagnostic. The absolute Q/Q_{exp} value could be influenced by the unknown modelled uncertainties, wrongly chosen number of factors, etc., and consequently, monitoring how the Q/Q_{exp} varies among different iterations could assist in assessing the optimal solution.[6]

Ions with signal-to-noise ratio (S/N) lower than 0.2 were removed, and the ions with S/N between 0.2 and 2 were downweighed by increasing the estimated error values.[5,7] The PMF solutions with one to ten factors are evaluated in this study. To further explore how rotation and the random starting point would influence the uncertainty of the solutions, different FPeak (from −1 to +1, step: 0.1), and SEED values (from 1 to 10, step: 1) were adapted, as described by Zhang et al.[8]

3. Detailed analysis procedures of ME-2

As mentioned in the main text section 2.3, ME-2 allows for solution of S1 while using a prior information about component mass spectra or time series. In this work we utilize a reference input profile for the PS mass spectrum with a scalar *a* value that determines the extent to which the derived factor profile could vary from the input spectrum profile.[6,9] The *a* value spans from 0 to 1 with an increment of 0.1, while the lower the *a* value, the more rigorous the constraint is. For example, if $a = 0.1$, for the profile of the extracted factor, the intensity of all the ions could vary as much as ± 10% compared with the input reference spectrum. The ME-2 with the *a* value approach could provide a more complete exploration of the rotational ambiguity of the solution space.[6]

4. The relative ionization efficiency (RIE) of polystyrene nanoplastic particles

To develop the calibration curve of pure polystyrene (PS) nanoplastic particles (NPP), a mixing condensation particle counter (CPC, Model 1720, Brechtel) was deployed together with AMS. The aerosols generated are split into two lines for AMS and the CPC, and the detected ion rate in Hz (I) from AMS could be calibrated with the CPC measurements.[10,11] The AMS ion signal was compared with the input number of molecules derived from the CPC particle number concentration and an assumed collection efficiency of 1 to obtain the ionization efficiency.

With ammonium nitrate as the standard, the relative ionization efficiency (RIE) for PS particles can be derived by comparing the ionization efficiency of the PS and ammonium nitrate. The detailed equation to calculate RIE is described in Eq. S1, Eq. S2, and Eq. S3 below:[10]

$$C_s = \frac{10^{12} MW_s}{IE_s Q N_A} \sum_{all\ i} I_{s,i} \quad \text{(S 1)}$$

$$\frac{IE_s}{MW_s} = RIE_s \frac{IE_{NO_3}}{MW_{NO_3}} \quad \text{(S2)}$$

$$C_s = \frac{10^{12} MW_{NO_3}}{RIE_s IE_{NO_3} Q N_A} \sum_{all\ i} I_{s,i} \quad \text{(S3)}$$

where the C_s is the mass concentration of the PS particles in this study, Q is the volumetric sample flow rate in $cm^3\ s^{-1}$, N_A is Avogadro number, and $\sum_{all\ i} I_{s,i}$ is the summation

of the detected ion rate in Hz for all fragment ions. IE_s and MW_s are the ionization efficiency and molecular weight of the PS particles, respectively, and the IE_{NO_3} and MW_{NO_3} represent the ionization efficiency and molecular weight of nitrate, respectively. The molecular weight of polystyrene cancels out by combining Eqns. (S1) and (S2) to derive Eqns. (S3).

To further test the reproducibility of the decomposition products of PS NPPs, the correlation values of the five mass spectra of PS NPPs collected during the five points calibration was calculated and plotted in Figure S4 below, and it shows nearly identical mass spectra. Besides, during the calibration experiment, the background blank samples were tested. Below in Figure S5, S6, S7 show the AMS mass spectra collected during the nitrate calibration, filtering period and the time series of tracer ions during the filtering period, which are 16 and 14 times lower than ambient-derived concentrations, respectively.

5. The comparison of PS NPPs quantification with the AMS and pyrolysis-GC/MS

To further validate the quantification of PS NPPs with the AMS, the pyrolysis GC-MS analysis was also conducted following the established method.[12] PS NPPs standard were collected by the AMS and onto a glass fiber filter (Cytiva, 0.7 µm particle retention) simultaneously with 2 liter per minute flow rate for 77 minutes.

The pyrolysis GC-MS method was calibrated with the mass of PS NPP standards ranging from 0.5 to 5 µg with the method established in previous study.[12] Briefly, the standards were first injected in the pyrolysis chamber. Pyrolysis was performed at 700 °C for 1 min, and thermodesorption was performed at 300 °C for 1 min. Gas phase pyrolysis products were injected directly into a Shimadzu GCMS-QP2020NX (Shimadzu Corporation, Kyoto, Japan) equipped with an RTX-1 capillarity column (30 m, 0.25 mm i.d., 25 µm film thickness). Column flow was 1.1 mL/min with helium as the carrier gas at a split ratio of 5. The injection port temperature was set at 310 °C. GC oven started at 50 °C for 2 min and ramped to 180 °C at 15 °C/min, increased to 310 °C at 5 °C /min, and hold at 310 °C for 44 mins. Mass spectrometry

scanned from 50 to 600 m/z range with ion source temperature at 200 °C and interface temperature at 280 °C. As shown below in Figure S8, the peak (~5 min) on the total ion chromogram (TIC) shows mass pattern matching to Styrene, a monomer of PS.[13] The instrument and membrane filter blanks do not show this PS peak on their chromogram. The areas of the PS peak (~5 min) on the TIC showed a linear relationship with the spiking masses, ($R^2 > 0.999$). The linear relationship indicates the feasibility of quantification for PS MPs in test samples.

Two replicated sets of 16 circular pieces (1.9 mm diameter) with a total area of 0.454 cm^2 were cropped from the sample filter. Given that the effective filter area is 7.55 cm^2, the mass of PS in each set of 16 circular pieces was projected to the mass of PS in the whole sampling filter by multiplying 16.63 (the ratio of the sample area to the measured area). Each set of 16 circular filter pieces was analyzed on a pyrolysis probe (CDS 6200, CDS Analytical, LLC. Oxford, PA, USA) with the same pyrolysis and GC/MS methods from above. The mass of PS NPPs in the 16 pieces of filter circles was estimated to be 0.57µg, which corresponding to 9.45 µg of PS collected on the filter and 61.36 µg/m^3 in the air sample measured by Pyrolysis-GC/MS. The mass concentration during the sampling time quantified by the AMS was 60.78 µg/m^3. The difference of PS concentration quantified by the AMS and the Pyrolysis-GC/MS is shown to be 2%, further validating an accurate quantification of PS NPPs with the AMS.

6. The second ambient sampling

The time series data for organic aerosols during the *ambient2* sampling period is shown in Figure S14. Applying similar analysis procedures to *ambient1*, the best ME-2 solution for *ambient2* sampling period has 3 factors, and they are identified as constrained PS factor, more-oxidized OOA (MO-OOA), and hydrocarbon like organic aerosols (HOA). Again, the MO-OOA factor has a higher f_{44} and O:C, and the HOA factor contains ions that have been identified as markers of fresh fossil fuel combustion, including $C_nH_{2n-1}^+$ and $C_nH_{2n+1}^+$.[14, 15]

7. ME-2 analysis of synthetic data matrix

To further validate the ability of ME-2 to quantify the concentration of ambient PS NPPs, the organic data matrix was modified before reapplying the ME-2 analysis. First, all the ME-2 derived factors were summed up except the extracted PS factor. The ME-2 analysis with the synthetic organic data matrix as input did not extract any meaningful PS factor, as the 10-minute moving average concentration has always been lower than the detection limit (12 ng/m^3 for 10 minutes). Second, the PS section in the original organic matrix was substituted with a fraction of the PS factor extracted by the original ME-2 analysis. 50%, 10%, 5%, and 2% of the original PS factor were added to the organic matrix generated by summing all factors except the original PS factor, and the ME-2 analysis was reapplied. The averaged PS concentration derived from the ME-2 analysis with the synthetic data matrix during the intentional injection period was 51%, 9%, 4%, and 2% of the original PS factor derived with the actual ambient data matrix with their absolute mass loading in the table S1. The additional ME-2 analysis successfully extracted a PS factor of around 16 ng/m^3, which is lower than the averaged concentration derived by the ME-2 for ambient sampling (30 $\pm$ 20 ng/m^3). Such results further validate the quantification of ambient PS NPPs with AMS and ME-2 analysis.

8. The concentration of airborne microplastic particles from literatures

In previous studies, the concentration of atmospheric microplastic particles was reported in various units across different geographical areas. Herein, this section illustrates the conversion of the previously reported concentrations into ng/m^3, which is the unit of measurement utilized in the present study, to provide a uniform basis for comparison. The majority of prior research quantified the microplastics in terms of number concentration, i.e. count per cubic meter.[16,17,18,19] By assuming a sphere for the collected microplastics, with the density and the midpoint of the reported size, the mass concentration of microplastics are derived from the number concentration. Another study reported the concentration of

microplastics as a percentage of the total particles collected,[20] and considering our sampling site, a total organic concentration of 6 μg/m^3 , which is the averaged concentration measured during the sampling, is adopted for the derivation in the present study.

9. Concentration of PS nanoplastic particles derived from the tracer ions

As described in section 3.4, to further validate the feasibility of using tracer ions to derive the abundance of PS nanoplastic particles, the concentrations from ME-2 and tracer ions are assessed. The concentration is calculated based on the normalized intensity of the tracer ion among the mass spectrum of the pure PS particle standard with the Eq. S4 below:

$$[PS] = [C_8H_8^+]/0.06 \qquad (S4)$$

where [PS] is the concentration of PS nanoplastic particles in μg/m^3, [$C_8H_8^+$] is the concentration of $C_8H_8^+$ in μg/m^3, and 0.06 is the normalized intensity of $C_8H_8^+$ for the mass spectrum of PS NPP standard.

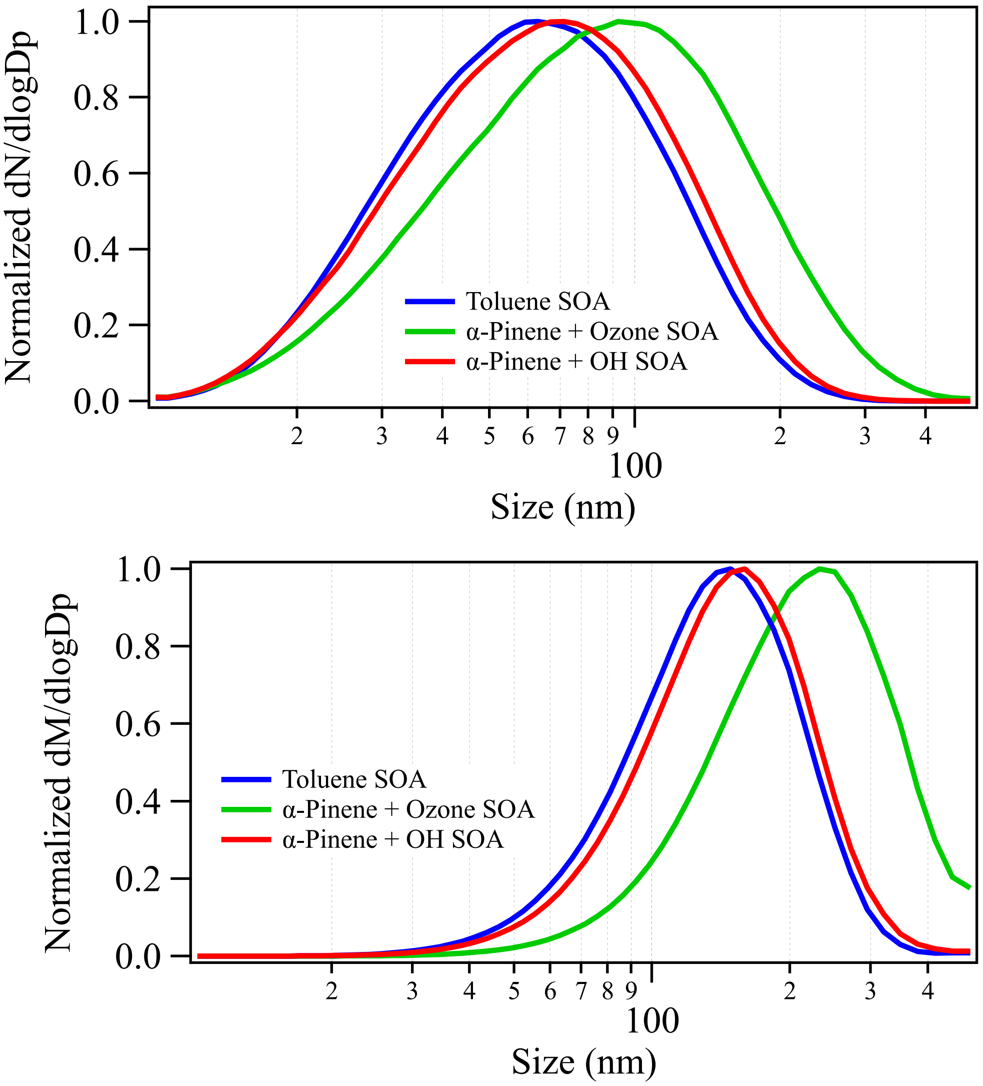

Figure S1: The normalized number and mass distribution of self-nucleated SOAs generated through PAM.

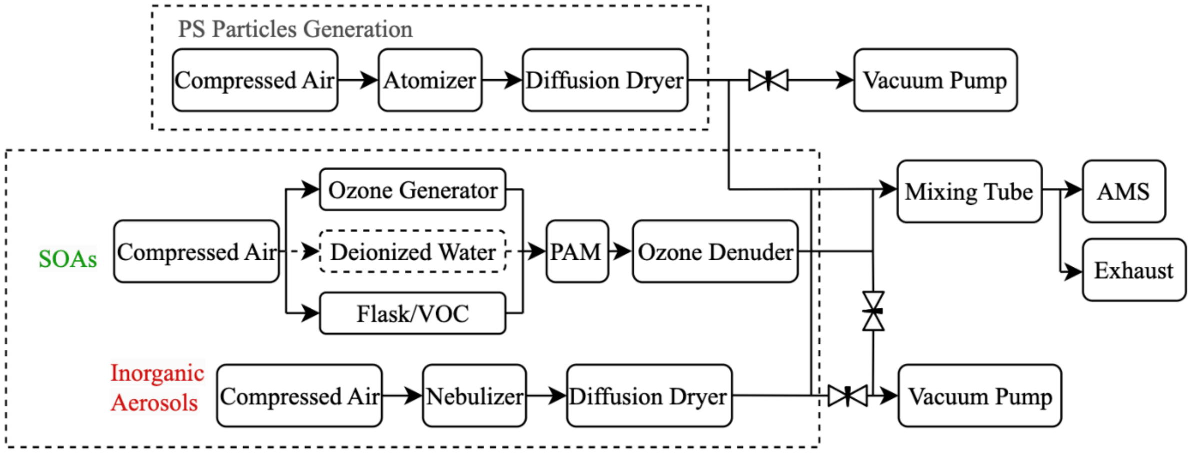

Figure S2. Experiments setup for the mixing of PS particles and SOAs/inorganic aerosols. The top part of the setup demonstrated the PS particles generation, and the bottom part shows the SOAs and inorganic aerosols generation. For the SOAs generation, deionized water line is only used during the OH radical oxidation pathway, whereas this line is replaced by pure air during the ozonolysis pathway.

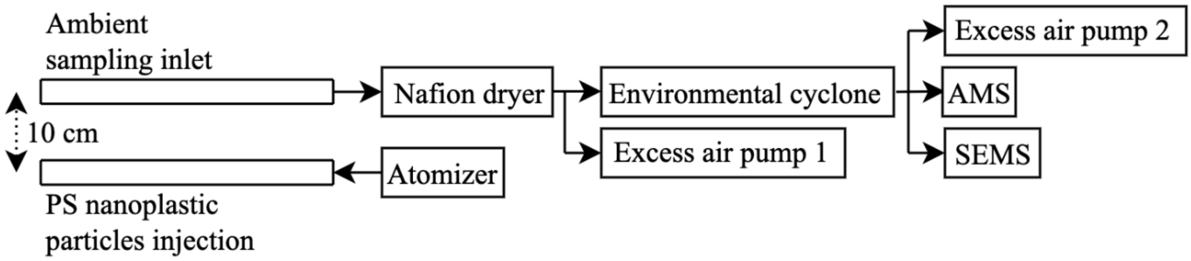

Figure S3. Schematic of ambient sampling setup. The excess air pump 1 drew 5 LPM of air to reduce wall loss of the submicron particles. The excess air pump 2 drew 1.6 LPM of air, to ensure the total flow passing through the environmental cyclone to be 2 liter per minute with a size cut of 1 μm.

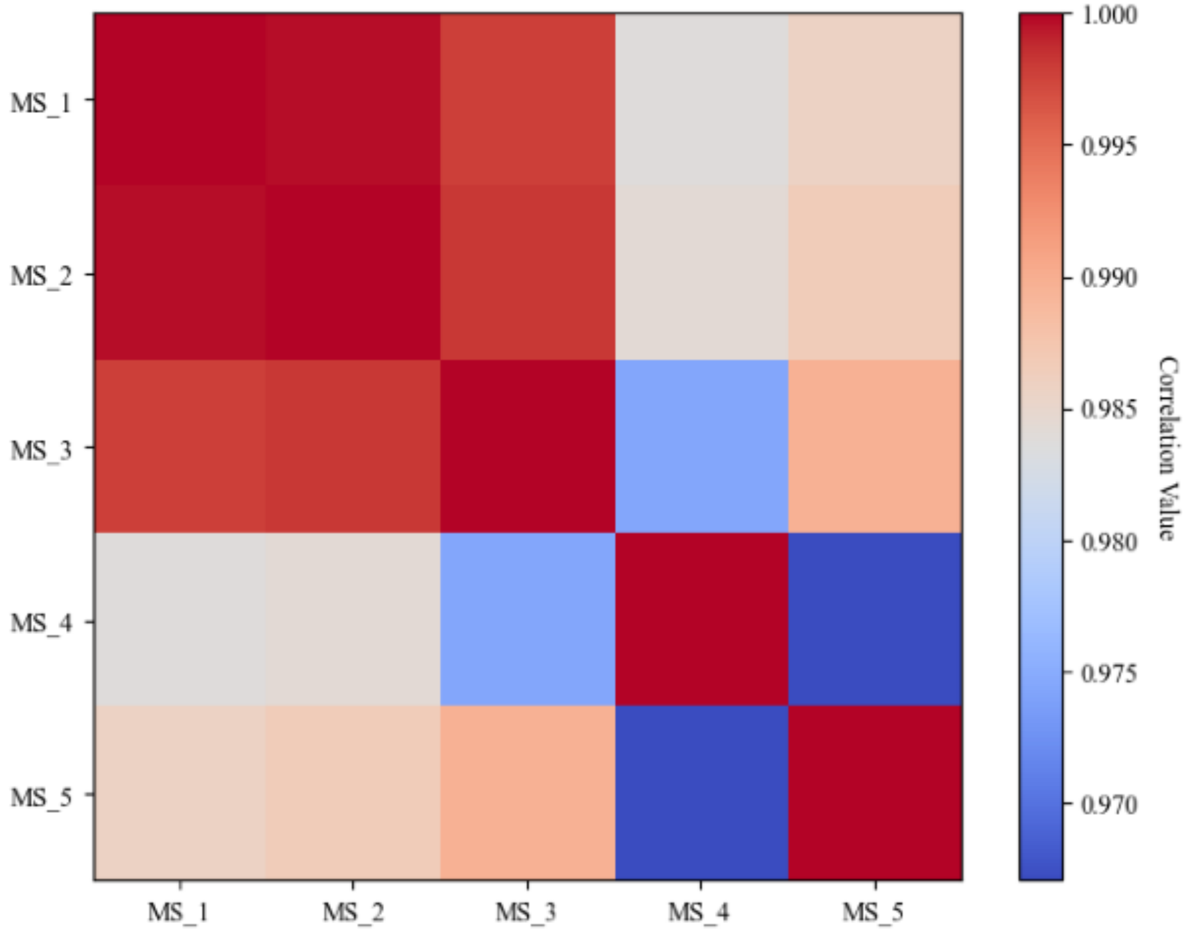

Figure S4. The Pearson's correlation heatmap of the five mass spectra of PS NPPs collected during the five-point calibration (MS_1 to MS_5).

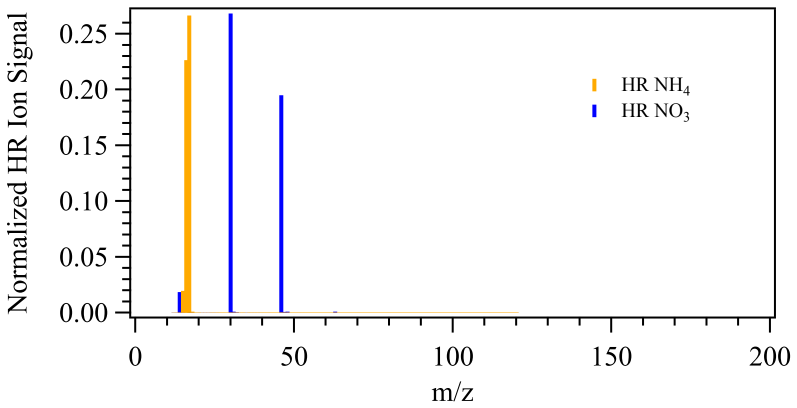

Figure S5. The mass spectrum of ammonium nitrate collected by the HR-ToF-AMS during the calibration procedures.

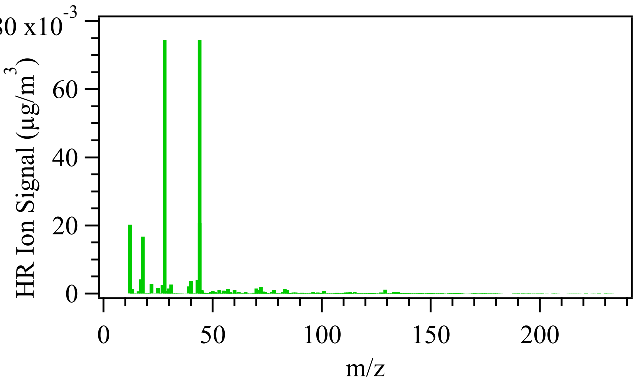

Figure S6. The mass spectrum collected from the HR-ToF-AMS when there was a filter installed at the inlet.

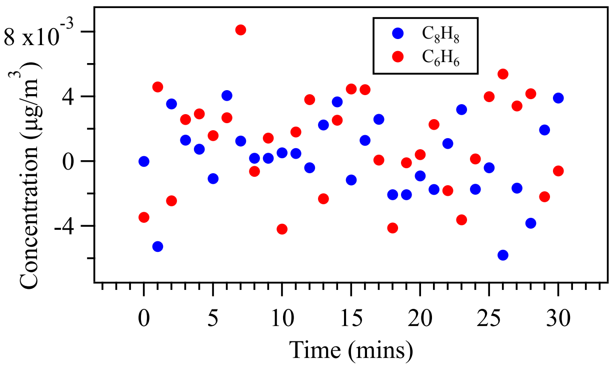

Figure S7. The derived concentrations of the tracer ions of PS NPPs when a filter was installed at the inlet of the HR-ToF-AMS.

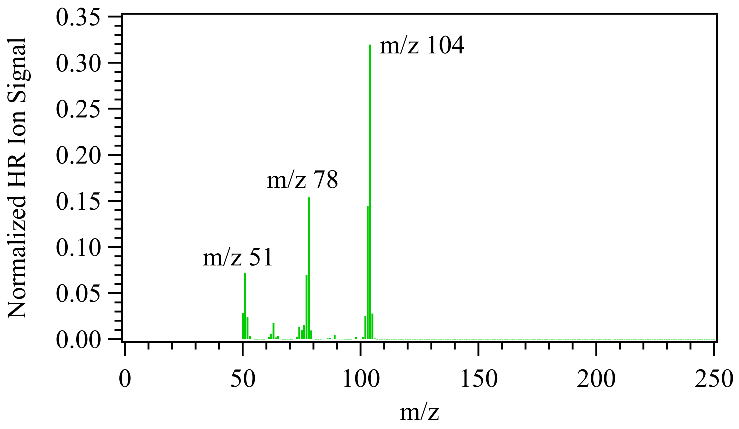

Figure S8. Pyrolysis-GC/MS mass spectrum for pure PS NPPs.

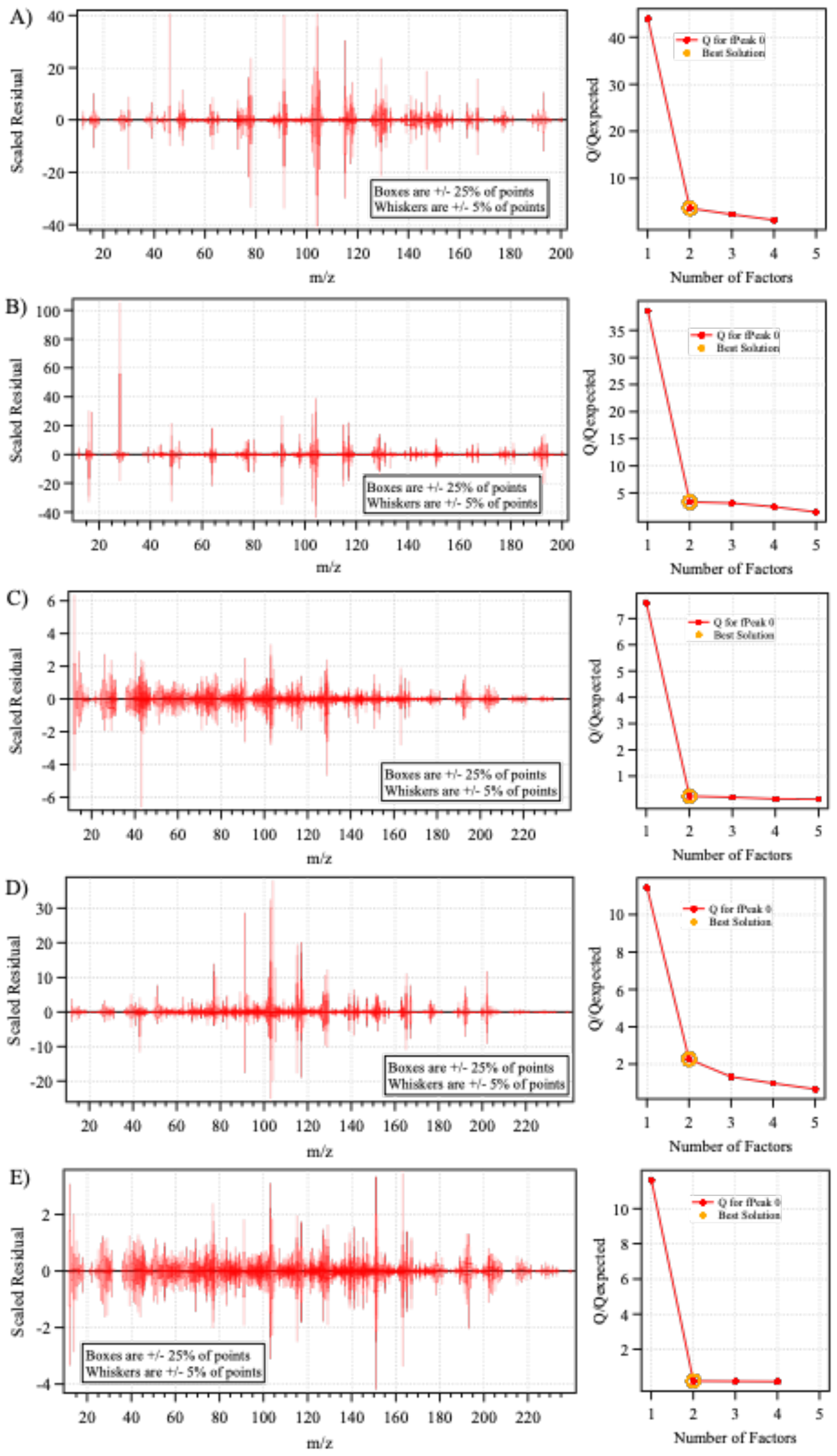

Figure S9. The scaled residual for the HR ions with two factors (right) and Q/Q_{exp} for the solution with different number of factors for different aerosol mixtures. A) ammonium

nitrate; B) ammonium sulfate; C) toluene with ozonolysis; D) α-pinene with ozonolysis and OH oxidation; E) α-pinene with ozonolysis.

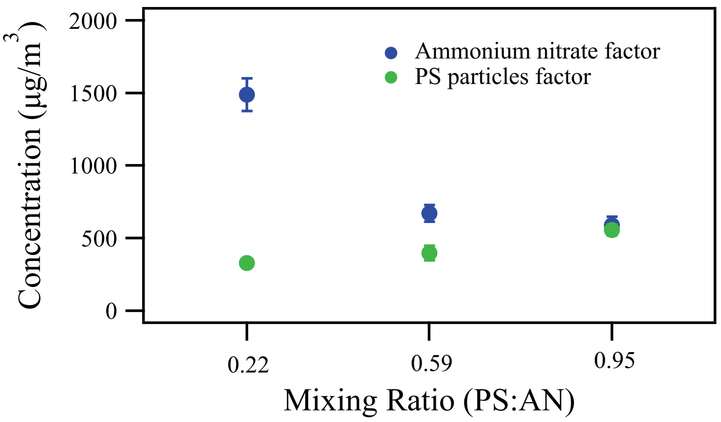

Figure S10. The time series of the two factors extracted by PMF for the mixture of PS nanoplastic particles and ammonium nitrate inorganic aerosols.

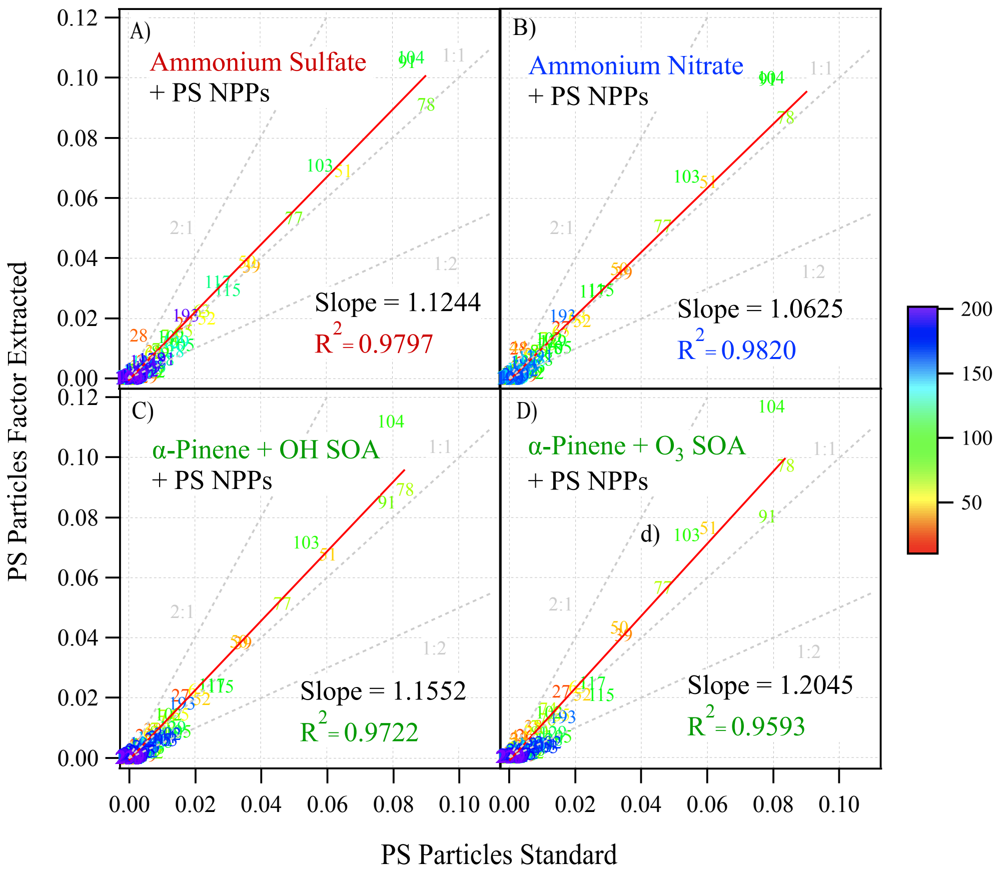

Figure S11. Scatter plots of normalized HR organic ion signal of PS particles from the factor extracted from PMF (y axis) of the four binary mixture systems versus the pure PS standard (x axis). The four binary mixtures are PS particles mixed with particles comprised of A) ammonium sulfate; B) ammonium nitrate; C) *α*-pinene SOA from OH oxidation and ozonolysis; D) *α*-pinene SOA from ozonolysis alone. The color bar represents the m/z from the mass spectrum in the order from low to high values.

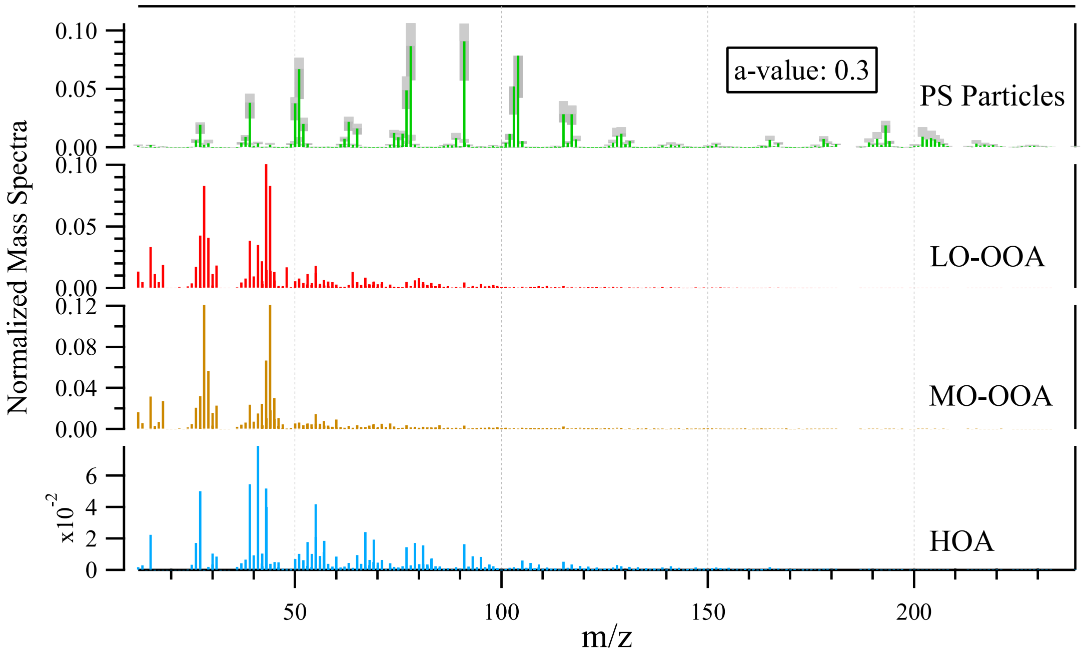

Figure S12. The normalized mass spectra (pr nor) of each factor identified during ambient1 from the ME-2 analysis. The grey shaded areas denote the uncertainty of the mass spectra with an *a*-value = 0.3.

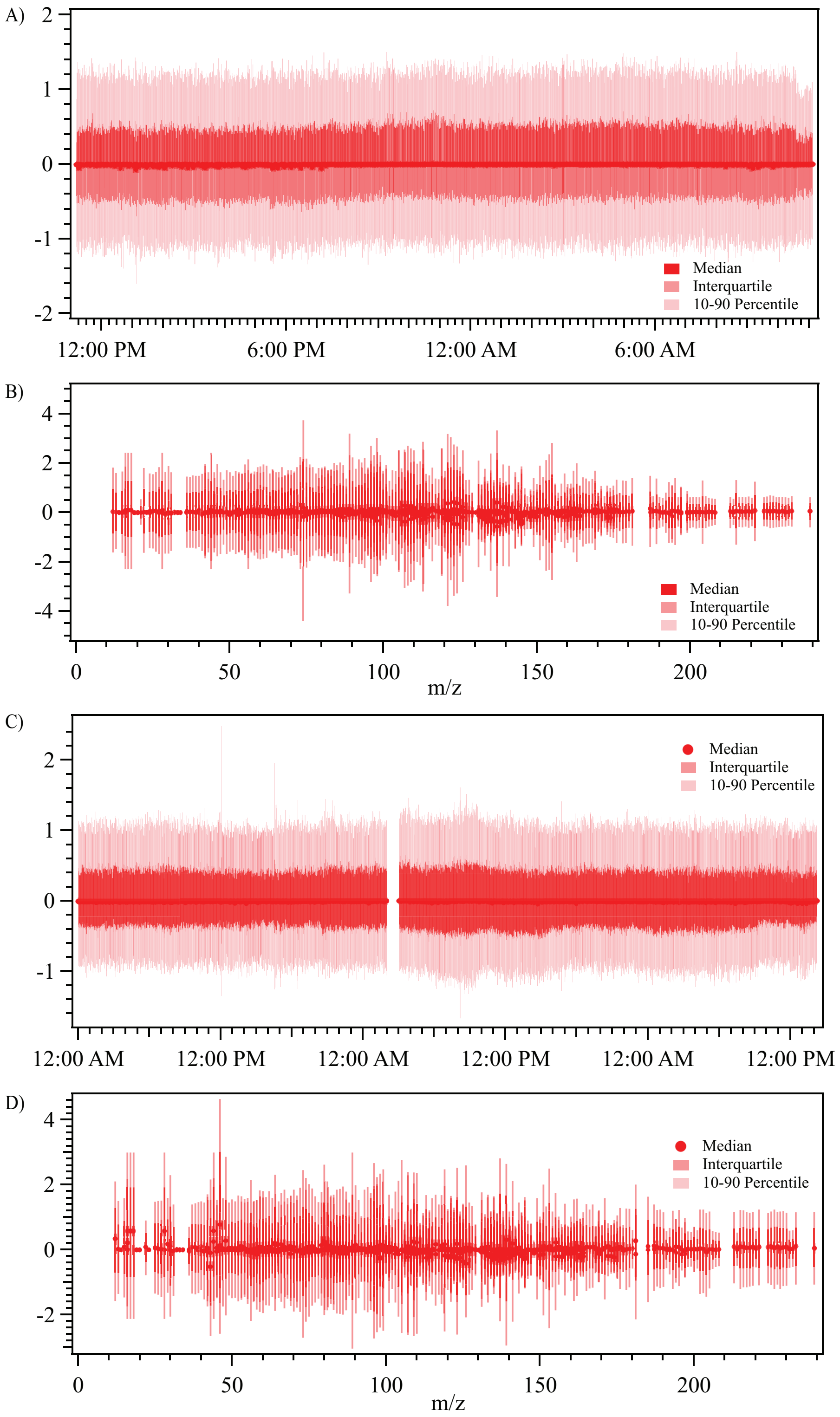

Figure S13. The scaled residual for ambient samplings among the sampling time (A, C) and mass spectrum (B, D) to demonstrate optimum solution is achieved with minimum residual. While A and B are for *ambient1* and C and D are for *ambient2*.

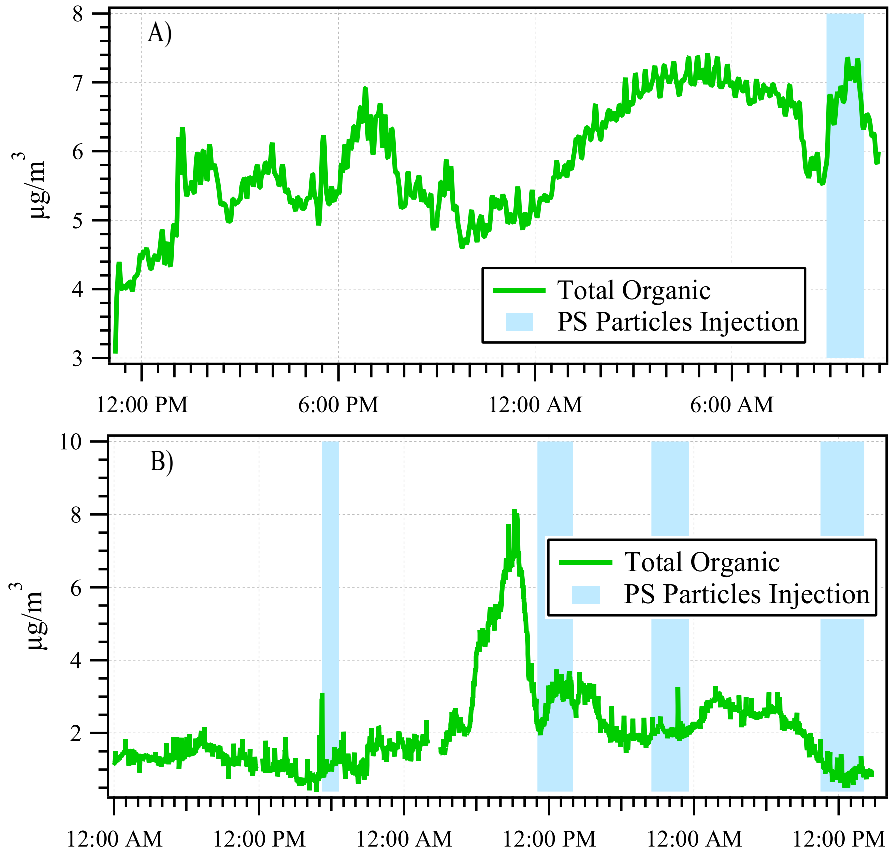

Figure S14. The time series of total organic concentration during *ambient1* (A) and *ambient2* (B) sampling.

Table S1. The ratio of the PS factor added to the synthetic organic data matrix for ME-2 analysis to the total PS factor derived from the original ME-2 analysis, the ratio of the PS factor derived from the corresponding ME-2 analysis to the total PS factor derived from the original ME-2 analysis, and the averaged mass loading of the PS factor derived from the corresponding ME-2 analysis during the intentional injection period.

Fraction of the PS factor added	Fraction of the PS factor derived	Averaged mass loading (ng/m^3)
100%	NA	654
50%	51%	333
10%	9%	61.5
5%	4%	26.9
2%	2%	16.2

Table S2. The averaged mass concentration and standard deviation of PS NPPs during specific period and their corresponding p value from t-test. The 4-6 pm indicate the background period, and the rest are the color-coded period in Figure 4A.

Time	4-6 pm	7-9 pm	9-11 pm	2-5 am	5-7 am
Mean Conc. (μg/m^3)	1.55×10^{-2}	1.78×10^{-2}	4.08×10^{-2}	4.05×10^{-2}	4.17×10^{-2}
SD	4.79×10^{-3}	7.27×10^{-3}	8.31×10^{-3}	7.80×10^{-3}	5.96×10^{-3}
p value	NA	4.44×10^{-3}	1.54×10^{-76}	2.34×10^{-79}	8.66×10^{-98}

References

(1) Drewnick, F.; Hings, S. S.; Alfarra, M. R.; Prevot, A. S. H.; Borrmann, S. Aerosol quantification with the Aerodyne Aerosol Mass Spectrometer: detection limits and ionizer background effects. *Atmos Meas Tech* **2009**, *2* (1), 33-46.

(2) Lambe, A. T.; Ahern, A. T.; Williams, L. R.; Slowik, J. G.; Wong, J. P. S.; Abbatt, J. P. D.; Brune, W. H.; Ng, N. L.; Wright, J. P.; Croasdale, D. R.; Worsnop, D. R.; Davidovits, P.; Onasch, T. B. Characterization of aerosol photooxidation flow reactors: heterogeneous oxidation, secondary organic aerosol formation and cloud condensation nuclei activity measurements. *Atmos Meas Tech* **2011**, *4* (3), 445-461.

(3) Lambe, A.; Massoli, P.; Zhang, X.; Canagaratna, M.; Nowak, J.; Daube, C.; Yan, C.; Nie, W.; Onasch, T.; Jayne, J.; Kolb, C.; Davidovits, P.; Worsnop, D.; Brune, W. Controlled nitric oxide production via $O(^1D) + N_2O$ reactions for use in oxidation flow reactor studies. *Atmos. Meas. Tech.* **2017**, *10* (6), 2283-2298.

(4) Paatero, P.; Tapper, U. Positive Matrix Factorization - a Nonnegative Factor Model with Optimal Utilization of Error-Estimates of Data Values. *Environmetrics* **1994**, *5* (2), 111-126.

(5) Ulbrich, I. M.; Canagaratna, M. R.; Zhang, Q.; Worsnop, D. R.; Jimenez, J. L. Interpretation of organic components from Positive Matrix Factorization of aerosol mass spectrometric data. *Atmos Chem Phys* **2009**, *9* (9), 2891-2918.

(6) Canonaco, F.; Crippa, M.; Slowik, J. G.; Baltensperger, U.; Prevot, A. S. H. SoFi, an IGOR-based interface for the efficient use of the generalized multilinear engine (ME-2) for the source apportionment: ME-2 application to aerosol mass spectrometer data. *Atmos Meas Tech* **2013**, *6* (12), 3649-3661.

(7) Paatero, P.; Hopke, P. K. Discarding or downweighting high-noise variables in factor analytic models. *Analytica Chimica Acta* **2003**, *490* (1), 277-289.

(8) Zhang, Q.; Jimenez, J. L.; Canagaratna, M. R.; Ulbrich, I. M.; Ng, N. L.; Worsnop, D. R.; Sun, Y. L. Understanding atmospheric organic aerosols via factor analysis of aerosol mass spectrometry: a review. *Anal Bioanal Chem* **2011**, *401* (10), 3045-3067.

(9) Paatero, P. The Multilinear Engine—A Table-Driven, Least Squares Program for Solving Multilinear Problems, Including the n-Way Parallel Factor Analysis Model. *J Comput Graph Stat* **1999**, *8* (4), 854-888.

(10) Canagaratna, M. R.; Jayne, J. T.; Jimenez, J. L.; Allan, J. D.; Alfarra, M. R.; Zhang, Q.; Onasch, T. B.; Drewnick, F.; Coe, H.; Middlebrook, A.; Delia, A.; Williams, L. R.; Trimborn, A. M.; Northway, M. J.; DeCarlo, P. F.; Kolb, C. E.; Davidovits, P.; Worsnop, D. R. Chemical and microphysical characterization of ambient aerosols with the aerodyne aerosol mass spectrometer. *Mass Spectrom Rev* **2007**, *26* (2), 185-222.

(11) Jimenez, J. L.; Jayne, J. T.; Shi, Q.; Kolb, C. E.; Worsnop, D. R.; Yourshaw, I.; Seinfeld, J. H.; Flagan, R. C.; Zhang, X.; Smith, K. A.; Morris, J. W.; Davidovits, P. Ambient aerosol sampling using the Aerodyne Aerosol Mass Spectrometer. *J Geophys Res Atmos* **2003**, *108* (D7), 8425.

(12) Ter Halle, A.; Jeanneau, L.; Martignac, M.; Jardé, E.; Pedrono, B.; Brach, L.; Gigault, J. Nanoplastic in the North Atlantic Subtropical Gyre. *Environmental Science & Technology* **2017**, *51* (23), 13689-13697.

(13) Shin, T.; Hajima, O.; Chuichi, W.; Tsuge, S.; Ohtani, H.; Watanabe, C. Pyrograms and thermograms of 163 high polymers, and MS data of the major pyrolyzates. *Pyrolysis-GC/MS Data Book of Synthetic Polymers* **2011**, 7-335.

(14) Zhang, Q.; Alfarra, M. R.; Worsnop, D. R.; Allan, J. D.; Coe, H.; Canagaratna, M. R.; Jimenez, J. L. Deconvolution and quantification of hydrocarbon-like and oxygenated organic aerosols based on aerosol mass spectrometry. *Environ Sci Technol* **2005**, *39* (13), 4938-4952.

(15) Shah, R. U.; Robinson, E. S.; Gu, P.; Robinson, A. L.; Apte, J. S.; Presto, A. A. High-spatial-resolution mapping and source apportionment of aerosol composition in Oakland, California, using mobile aerosol mass spectrometry. *Atmos Chem Phys* **2018**, *18* (22), 16325-16344.

(16) Liu, K.; Wang, X.; Fang, T.; Xu, P.; Zhu, L.; Li, D. Source and potential risk assessment of suspended atmospheric microplastics in Shanghai. *Sci Total Environ* **2019**, *675*, 462-471.

(17) Asrin, N. R. N.; Dipareza, A. Microplastics in ambient air (case study: Urip Sumoharjo street and Mayjend Sungkono street of Surabaya City, Indonesia). *J Adv Res Appl Sci* **2019**, *6*, 54-57.

(18) Dehghani, S.; Moore, F.; Akhbarizadeh, R. Microplastic pollution in deposited urban dust, Tehran metropolis, Iran. *Environ Sci Pollut Res* **2017**, *24* (25), 20360-20371.

(19) Liu, K.; Wu, T.; Wang, X.; Song, Z.; Zong, C.; Wei, N.; Li, D. Consistent Transport of Terrestrial Microplastics to the Ocean through Atmosphere. *Environ Sci Technol* **2019**, *53* (18), 10612-10619.

(20) Liu, C.; Li, J.; Zhang, Y.; Wang, L.; Deng, J.; Gao, Y.; Yu, L.; Zhang, J.; Sun, H. Widespread distribution of PET and PC microplastics in dust in urban China and their estimated human exposure. *Environ Int* **2019**, *128*, 116-124.
